# Genetically engineered bacterium: Principles, practices, and prospects

**DOI:** 10.3389/fmicb.2022.997587

**Published:** 2022-10-13

**Authors:** Yiting Liu, Jing Feng, Hangcheng Pan, Xiuwei Zhang, Yunlei Zhang

**Affiliations:** ^1^Department of Respiratory and Critical Care Medicine, The Affiliated Jiangning Hospital of Nanjing Medical University, Nanjing, China; ^2^Department of Biomedical Engineering, School of Biomedical Engineering and Informatics, Nanjing Medical University, Nanjing, China; ^3^Central Laboratory, Translational Medicine Research Center, The Affiliated Jiangning Hospital of Nanjing Medical University, Nanjing, China

**Keywords:** bacteriotherapy, genetically engineered bacterium, clinical application, synthetic biotechnology, heterologous expression

## Abstract

Advances in synthetic biology and the clinical application of bacteriotherapy enable the use of genetically engineered bacteria (GEB) to combat various diseases. GEB act as a small ‘machine factory’ in the intestine or other tissues to continuously produce heterologous proteins or molecular compounds and, thus, diagnose or cure disease or work as an adjuvant reagent for disease treatment by regulating the immune system. Although the achievements of GEBs in the treatment or adjuvant therapy of diseases are promising, the practical implementation of this new therapeutic modality remains a grand challenge, especially at the initial stage. In this review, we introduce the development of GEBs and their advantages in disease management, summarize the latest research advances in microbial genetic techniques, and discuss their administration routes, performance indicators and the limitations of GEBs used as platforms for disease management. We also present several examples of GEB applications in the treatment of cancers and metabolic diseases and further highlight their great potential for clinical application in the near future.

## Introduction

Despite the success of modern medical technologies in the prevention and treatment of most human diseases, the rapid increase in antibiotic-resistant microorganisms (Cassini et al., 2019) and chronic patient populations (Bernell and Howard, 2016; Bray et al., 2018; Blüher, 2019; Khan et al., 2020; Powell-Wiley et al., 2021), adverse effects caused by chemical drugs, exorbitant medical costs (Gheorghe et al., 2018; Lentz et al., 2019), cancers and other incurable diseases urgently require highly economic, convenient and efficient methods for addressing these issues. Increasing evidence demonstrates the links between the dysfunction of the human microbial community and the onset and development of many human diseases (Gilbert et al., 2016; Dang and Marsland, 2019), signifying the potential use of microorganisms as an alternative strategy to conquer these issues (Mazhar et al., 2020). Remarkably, bacteria account for over 90% of intestinal microbes, and advances in synthetic biology enable the precise manipulation of bacteria for diverse purposes (Lawson et al., 2019). For instance, technical improvement in Clustered regularly interspaced short palindromic repeats-cas9 (CRISPR-cas9; Li et al., 2020; Wang et al., 2020) and molecular biological methods (e.g., gene synthesis, DNA/RNA sequence, DNA transfection, clone techniques of large DNA fragment, etc.; Tavanti, 2022) greatly contribute to generating various engineered bacteria for producing many proteins and molecular compounds that originate from uncultivated microorganisms, fungi, archaea, microeukaryotes and eukaryotes (Çelik and Çalık, 2012; Gupta and Shukla, 2016; Charbonneau et al., 2020). Mounting evidence demonstrates that genetically engineered bacteria (GEB) can be orally or intravenously administered in clinical trials to cure different diseases (Liu et al., 2016; Zhang et al., 2018). Due to their special colonization ability in solid tumors, intestinal tracts, respiratory tracts, genital tracts, and the oral cavity, GEBs perform their activities by supplying active molecules, interfering with immune cells, restraining pathogenic bacteria, or killing tumor cells by expressing foreign genes or enhancing endogenous gene expression, thus preventing, diagnosing or curing diseases (Fan et al., 2019). Here, we systemically summarize the recent advances in bacteriotherapy, which uses GEBs as the main body, including the introduction of genetically engineered bacteria, engineering techniques, administration strategies, performance indicators, and biological safety. Finally, we describe the preclinical and clinical applications of GEBs and several probiotics in the treatment of cancers and metabolic diseases and discuss their limitations and prospects.

## GEB in disease management

### GEB

**Figure 1:**
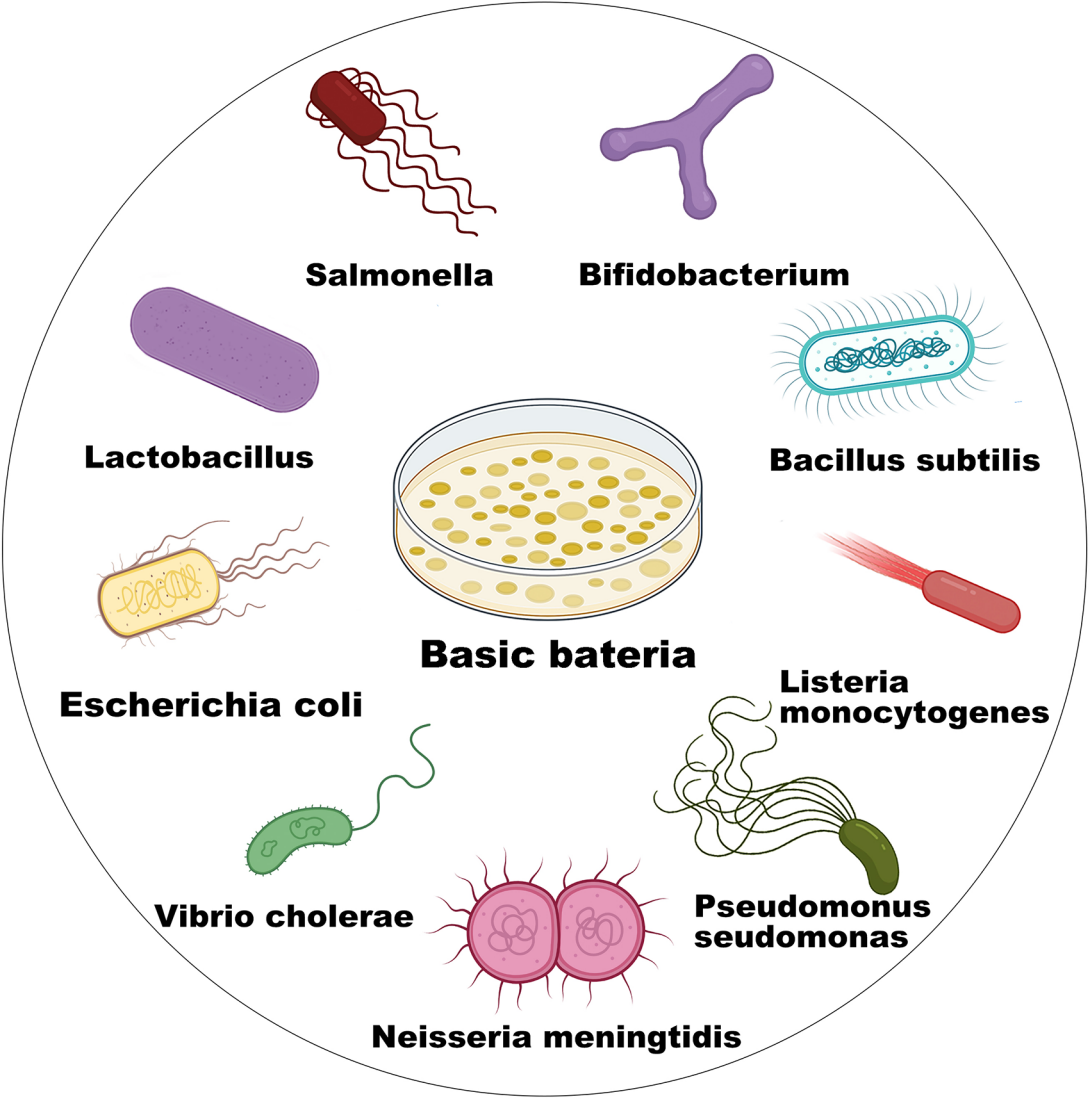
Microbial chassis for GEB construction.

A GEB is defined as a bacterium with the ability to efficiently express heterologous proteins or molecular compounds for a specific purpose after genetic engineering. As early as the 19th century, Coley revealed the therapeutic effects of inactivated bacterial mixtures in sarcoma therapy (Coley, 1893). By the end of the 20th century, genetic engineering techniques have been widely used to modify bacteria to obtain the expected compounds. After decades of effort, numerous GEBs have been established for various applications in the food industry, disease treatment, chemical synthesis, environmental protection, etc. Notably, *E. coli* (Michael Schultz, 2008; He et al., 2017; Christofi et al., 2019; Terol et al., 2021), *Lactobacillus* (Yang et al., 2015; Lin et al., 2016; Chen et al., 2017; Oh et al., 2020), *Salmonella* (Chirullo et al., 2015; Zheng and Min, 2016; Li et al., 2017; Lim et al., 2017; Kawaguchi et al., 2018) are the most popular bacteria used as chassis tools for constructing different GEBs. In particular, more than 50 bacterial species, such as *Bifidobacterium* (Liu et al., 2018), *Bacillus subtilis* (Westbrook et al., 2016), *Listeria monocytogenes* (Selvanesan et al., 2022), and *Lactobacillus brevis* CD2 (He et al., 2017; Alfano et al., 2020), are being used in health care and scientific research (Figure 1).

### Advantages of GEBs in disease management

To date, GEBs have made great achievements in the management of various diseases, such as infectious diseases, antibiotic-related diarrhea, allergies, and metabolic syndromes, in health care for daily life (Ma et al., 2017; Mazhar et al., 2020). Importantly, GEBs have more systematic and comprehensive therapeutic effects than traditional methods in the prevention and treatment of some diseases (Duan et al., 2015; Isabella et al., 2018; Kurtz et al., 2019) partly because the colonized microorganisms in the body are almost equal to tiny living factories that can autonomously reduplicate, detect abnormal homeostasis, produce therapeutic agents, and initiate self-destruction at a defined time (Pedrolli et al., 2019). Accordingly, GEBs have promising potential to be used at different stages of disease for different purposes. Genetically engineered bacterial vaccines, such as *Salmonella*, *Vibrio cholera*, *Listeria*, and *Neisseria meningitidis*, are capable of activating *in vivo* immune protective responses by introducing some effective antigens into low- or nonvirulence bacteria (Le et al., 2015; Herzog, 2016; Sharma et al., 2020). Using cholera as an example, the oral administration of attenuated *Haiti V* to rabbits efficiently restrained the colonization of wild *vibrio cholera* in the small intestine, thus reducing the incidence of *Vibrio cholera* infection (Hubbard et al., 2018; Satchell, 2018). Additionally, genetically engineered *Lactobacillus* succeeded in detecting *Vibrio cholerae* in stool samples *via* the specific phenomenon of “quorum sensing” in pathogenic microorganisms (Carignan et al., 2016; Mao et al., 2018). In cancer therapy, some bacteria, such as *Salmonella*, *Clostridium novyi-NT*, and *E. coli*, have a special ability to colonize solid tumors, enabling them to be excellent candidates for drug delivery and drug production (Wei et al., 2016). For example, transforming growth factor alpha-*pseudomonas* exotoxin-expressing *S. typhimurium* displayed significant inhibitory effects on the growth of CT26, MC38, and 4 T1 solid tumors (Lim et al., 2017). In melanoma therapy, recombinant *S. typhimurium* transfected with the interferon-gamma gene plasmid integrated into the N-terminal region (residues 1–160) of a surface immunogenic protein demonstrated obvious toxicity to cancer cells (Yoon et al., 2017). In addition, *S. typhimurium* loaded with CpG ODN and PD-1-siRNA induced innate immunity and inhibited PD-1 expression, thus killing cancer cells (Jia et al., 2021).

To date, the generally used GEBs are mainly confined to some intestinal diseases, such as inflammatory bowel disease (IBD; Saez-Lara et al., 2015) and cholera (Duan and March, 2010; Satchell, 2018). For instance, *Lactobacillus* transfected with interleukin 1 receptor antagonist was capable of reducing CD4^+^ IL-17A^+^ cells in mesenteric lymph nodes and blocking IL-1-cell signaling, thus alleviating the symptoms of acute colitis (Namai et al., 2020); *E. coli Nissle* 1917 (EcN) with the expression of cholera autoinducer 1 could restrain the virulence gene expression of *Vibrio cholera* and, thus, reduce its colonization in the gut (Duan and March, 2010). Essentially, the advantages of GEBs could be briefly summarized as follows: reduced cost of health care because of manufacturing scale-up and long-term effects of GEBs in colonization sites; decreased adverse effects, especially when they are orally administered; and for structurally unstable or environmentally sensitive compounds, free of drug purification and low-temperature storage. Notably, GEBs can produce multiple foreign proteins or compounds in one strain instead of requiring several drugs to achieve synergistic treatments (Peters et al., 2019).

## Microbial genetic engineering

Microbial genetic engineering uses genetic operation tools to shear, splice, and integrate the target genes and then introduce them into chassis cells. Thus, the recombinant genes are transferred into the desired products or endow the bacteria with new phenotypes. Due to the great progress in sequencing technologies and bioinformatics, a growing body of functional genes and gene clusters from nonculturable microorganisms are being excavated. How to express functional genes or gene clusters in a chassis cell is becoming a new research hotspot in the study of GEBs.

Indeed, GEB construction mainly includes the following two stages: upstream (functional gene acquisition) and downstream (heterogeneous expression). The acquisition of target genes or large gene clusters is an important part of the upstream stage of constructing an expected GEB (Cobb and Zhao, 2012). Due to the national microbial genome projects launched in multiple countries, numerous potential genes are available to be used for fabricating various GEBs. In fact, the size of the targeted genes determines the choice of MGE for GEB construction. The acquirement and modification of small gene fragments (<10 kb) can be performed using the general or long PCR method or direct DNA synthesis and restriction enzyme digestion (Fahnøe and Bukh, 2019). However, when the gene size exceeds 50 kb, some recombination methods, such as CRISPR–Cas9 and the Red/ET recombination system, are the optimal methods to alter, replace, delete or add bases or gene fragments of plasmids or genomes (Li and Elledge, 2012; Luo et al., 2016; Alberti and Corre, 2019; Strain-Damerell et al., 2021). Specifically, CRISPR–Cas9 can edit bacterial DNA fragments up to 100 kb in a single step in which RNA-guided Cas9 nuclease targets and cleaves DNA fragments, and the final large gene fragments are assembled *via* Gibson assembly (Jiang et al., 2015).

**Figure 2:**
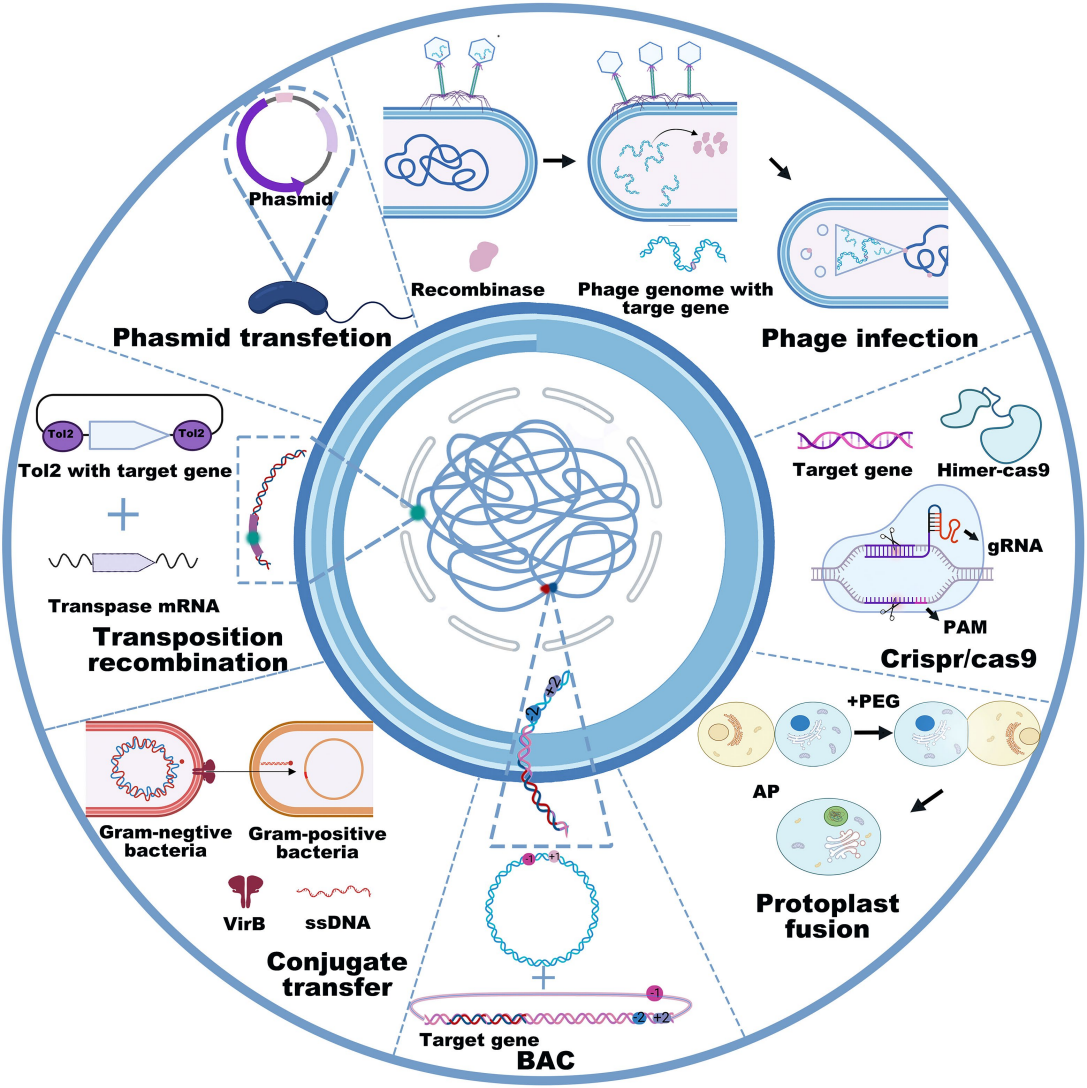
General used recombination technologies, including phage infection, phasmid transfection, transposition recombination, conjugate transfer, BAC, protoplast fusion, and CRISPR-Cas9 in counter-clockwise order.

Cloning target genes or gene clusters into bacterial chassis includes gene transfer and genetic recombination using the techniques of transfection, transduction, conjugative transfer, lysogenic conversion, and protoplast fusion. The DNA size and property of the bacterial chassis determine the transfer methods. Heat-shock and electroporation transfection are widely used to transfer plasmids in *E. coli*, *Salmonella*, *Bacillus thuringiensis*, *Pseudomonas aeruginosa*, etc. Conjugative transfer and protoplast fusion often involve the transfer of plasmids from donor bacteria to recipient bacteria (Mukai et al., 2020). For instance, the conjugative type IV secretion system is synergistic with DNA-processing machinery termed the “relaxosome,” and a large extracellular tube termed the “pilus” is capable of orchestrating directional conjugated plasmid transfer (Waksman, 2019). Additionally, homologous recombination technologies, mainly including homologous recombination, site-specific recombination, transposable recombination and the CRISPR–Cas9 technique, enable the direct integration of target genes into the host chromosome in an expected strain. The typical homologous recombination methods require more than 1 kb homologous sequences to realize the recombination of target genes into the chromosomal genome (Beumer et al., 2013). Due to CRISPR–Cas9 technology, DNA operation has become much more efficient and accurate, which greatly benefits the extraction and heterologous expression of gene clusters in chassis cells, especially of those with a size of over 50 kb. Transposition (Martínez-García and de Lorenzo, 2012) and homologous recombination (Wang et al., 2012; Davy et al., 2017) are also available to integrate exogenous DNA fragments into the bacterial genome. Remarkably, when selection marker or CRISPR editing alleles are not applicable, transposition is an optimal choice for integrating target genes into the bacterial genome (Jiang et al., 2013; Vo et al., 2021). To date, the generally used transposases mainly include sleeping beauty, piggyBac (Tschorn et al., 2020), Tol2 (Ni et al., 2016), Tn5 (Balasubramanian et al., 2021; Xu et al., 2021), Tn7 (Kaczmarska et al., 2022), and ICEBs1 (Ni et al., 2016; Peters et al., 2019; Strecker et al., 2019; Wu et al., 2021), all of which possess the ability to integrate small or large DNA fragments into bacterial genomes (Figure 2). However, the transposition efficiency slightly decreases as the size of the inserted DNA fragments increases (Kowalczykowski, 2015). Interestingly, several studies have shown that the synthetic Himar1 transposase-dead Cas9 fusion protein, which is characterized by DNA integration ability by Himar1 transposase (a Tn7-like transposon) and targeted localization by programmable dead Cas9, is capable of achieving targeted transposition under cell-free condition, thus avoiding the random insertion of transposons (Bhatt and Chalmers, 2019). This recombination method succeeded in accomplishing the transfer of transposons larger than 7 kb, the accuracy of which was as high as 80% (Chen and Wang, 2019). Similarly, another integrated system originating from Tn6677 transposase utilized a Tn7-like transposon and CRISPR protein to guide RNA-assisted targeted insertion of transposable factors, enabling the accurate insertion of over 10 kb DNA sequences into bacterial genomes (Nelson et al., 2021).

However, the plasmid carrying capacity and transposition method do not allow for operating large DNA fragments, especially when their sizes are larger than 100 kb (Hashimoto et al., 2015). To achieve the heterologous expression of large gene clusters, researchers are more inclined to use transformation-associated recombination (Bilyk et al., 2016; Zhang et al., 2019), bacterial artificial chromosomes (BAC; Huo et al., 2019; Hashimoto et al., 2020b), phage recombination systems (Oßwald et al., 2014; Nah et al., 2017), or integrase-mediated recombination systems (Du et al., 2015; Ke et al., 2021; Figure 2). For example， the BAC technique enabled the integration of over 181 kb DNA fragments into the genome of *Streptomyces lividans* TK23 (Hashimoto et al., 2020a). Concomitantly, the protoplast fusion method is also an option to acquire new functions for GEBs by integrating two bacterial genomes (Zhang et al., 2002). Although the insertion of foreign genes into the genome is more complex than the plasmid-based expression system, it possesses incomparable stability (Miyazaki and van der Meer, 2013; Pedrolli et al., 2019). Collectively, these methods, such as DNA extraction, site-specific mutation, gene insertion, gene deletion, and DNA transfection, have the potential to cater to most genetic manipulations in GEB construction, even when their size exceeds 100 kb. However, it is still a considerable challenge to establish GEBs for reliably and efficiently expressing foreign proteins or compounds mainly because interspecific differences disable the microbial chassis from expressing most foreign genes or gene clusters, even after codon optimization. Therefore, the principal contradiction of establishing GEBs is that gene sequencing reveals an increasing number of functional genes or gene clusters from microorganisms characterized by nonculture, a long growth cycle, or harsh culture, but not enough microbial chassis are available to express or deliver them for disease diagnosis and treatment.

## Administration route and performance indicators

### GEB administration route

The route of GEB administration depends on multiple factors, including the target tissues, disease types, properties of heterogeneous proteins or compounds, and chassis cells (Hosseinidoust et al., 2016). However, considering the potential pathogenicity of microorganisms, especially when administered *via* intravenous administration, the delivery route should be seriously considered at the initial stage of GEB construction in health care and disease management. Intensive clinical trials demonstrate that therapeutic and adverse effects are closely associated with drug delivery models (Ott et al., 2004). To date, the routes of GEB administration mainly included oral administration, intravenous injection, intratumoral injection, nasal administration, and subcutaneous injection.

Oral administration is the most widely used method in bacteriotherapy because of its simple operation, noninvasiveness, and wide applicability. However, the gastrointestinal tract is known to be a complex environment, including drastic pH changes among different organs [pH 1.0–2.5 in the stomach (Lund et al., 2014), pH 7–7.4 in the small intestine, and pH 6–6.7 in the colon (Kamada et al., 2013; Koziolek et al., 2015)] and differences in oxygen percentages (Zheng et al., 2015), nutrient enrichment, and flora diversity, which greatly affect GEB activity when orally administered (Sender et al., 2016). Notably, the survival rates of GEBs have a great influence on their therapeutic efficacies, but they do not exceed 50% when they are orally taken without extra protection, highlighting the key role of shielding bacteria from a complex gastrointestinal environment (Heavey et al., 2021). Indeed, the emergence of new technologies, such as polysaccharides (alginate, κ-carrageenan, locust bean gum, gellan gum, and xanthan gum; Ta et al., 2021), cationic liposomes (Chowdhuri et al., 2016), and mammalian cell membranes (Cao et al., 2019a), enable the isolation of bacteria from hostile environments. However, different packaging methods have different effects on the survival rates of GEBs after oral administration (Chowdhuri et al., 2016; Han et al., 2016; Sánchez et al., 2017; Cao et al., 2019b; Gharibzahedi and Smith, 2021). For instance, liposomal emulsified bacterial vaccines exhibited higher immune-boosting capacity and therapeutic efficiency than the uncoated vaccines in animal experiments (Naciute et al., 2021).

Comparatively, the intravenous administration of live bacteria only occurs in preclinical and clinical cancer therapy due to the high pathogenicity of the systematic administration of GEBs (Forbes et al., 2018). Additionally, nasal drip is another administration route of GEBs. Due to the specific safeguard function of nasal mucosa, intranasal vaccination becomes capable of activating local humoral and cellular immune responses at the entrance of the respiratory mucosa, distal mucosal sites, and their associated lymphoid tissues, thereby exerting prevention or therapeutic effects (Riese et al., 2014; Xu et al., 2021). Notably, bacterial administration *via* blood, nasal or intestinal routes is very beneficial for them to quickly reach disease sites, and thus, in most cases, their therapeutic effects are superior to those of oral administration (Wells and Mercenier, 2008; Pandey et al., 2022). However, some studies signified that the oral administration of GEBs was able to produce a higher immune response than intranasal immunization (Wan and Ping, 2021). The controversial issue is probably attributed to the use of distinct disease molds in these studies. In contrast to the treatment of enteric disease through oral administration, intranasal immunization is more suitable for the prevention of systemic allergy and airway inflammation (Sarate et al., 2019). In fact, various recombinant bacteria, such as *Streptococcus gordonii, Staphylococci,* and *Lactobacillus,* have shown great potential as active carriers of nasal vaccines (Mojgani et al., 2020; Dadar et al., 2021).

Furthermore, intratumoral injection is another key administration route for live GEBs to treat solid tumors. This method is greatly beneficial for reducing the potential systemic toxicity of live GEBs (Taniguchi et al., 2010). The intratumoral injected bacteria actively or passively colonize necrosis because of their homing instincts, chemotactic effects (Mirkhani et al., 2021), and cumulative effects (Ganai et al., 2011). Then, they activate immunogenicity and release toxic molecules to induce cell apoptosis and restrain tumor growth (Yaghoubi et al., 2020; Lin et al., 2021). However, this treatment may lead to highly malignant adverse effects because the biological toxicity caused by rapid and massive cell death in highly colonized bacterial tumors is extremely harmful to other normal organs and even induces a cytokine storm, thus leading to patient death (Karbach et al., 2012; Pandey et al., 2022). Therefore, the administration route is a key factor in GEB construction for different purposes.

### GEB performance indicators

To date, there are still no official standards for evaluating the efficacy of GEBs in disease diagnosis, prevention, or treatment. Increasing evidence has demonstrated that the intrinsic biological property, colonization ability, dose tolerance, and potential pathogenicity of GEBs all influence their activities. Establishing standard performance indicators plays a key role in advancing GEB clinical transition.

#### Colonization ability

GEB colonization ability refers to their survival and biological inheritance at the expected sites of disease after they enter the body and is an important indicator for evaluating GEB efficacy in disease management. Various techniques, mainly including tissue sectioning, fluorescent labeling, 16S rRNA sequencing, quantitative polymerase chain reaction, etc., have been developed to evaluate GEB conization ability (Cronin et al., 2012; Shen et al., 2015). Fluorescently labeled GEBs can be traced and recorded from the initial administration to fecal samples under a fluorescence microscope; sequencing technology could use 16S rRNA gene tags to obtain the current bacterial lineage in fecal samples, thus evaluating the colonization ability of GEBs by comparison with the initial fecal flora (Shen et al., 2015).

Recent studies have identified some bacteria with high intestinal colonization (Dosoky et al., 2020). For instance, *E. coli* NGF-1 has been found to colonize *in vivo* for up to 6 months in a stable and persistent state (Riglar et al., 2017). However, various external factors may influence the *in vivo* colonization ability of GEBs. The key influencing factor is the location of GEBs in the body because each bacterium in our body has a corresponding colonization area in which the microenvironment formed by long-period interactions with other bacteria and mammalian cells provides a safe place for them to proliferate with high genetic stability (O’Toole and Claesson, 2010; Zou et al., 2019; Tochitani, 2021). Therefore, the disease location in the body determines the species of chassis bacterium, thus enhancing their colonization and reducing the off-target effects of their secreted substances (Dosoky et al., 2020).

Indeed, some studies have taken advantage of organ-specific GEBs to exert their best therapeutic effects (Tarahomjoo, 2012), such as using colon-colonizing strains to treat ulcerative colitis (Conrad et al., 2014) and employing *Lactobacillus* bacteria, which colonize in the small intestine and colon, to treat Crohn’s disease (Donaldson et al., 2016). More importantly, the administration method also influences GEB colonization ability. The intravenous injected *Salmonella typhi* exhibited higher tumor colonization ability than the intraperitoneal administered ones. In addition, the tumor size and bacterial number affect GEB colonization in target sites (Mei et al., 2002). Currently, the studies investigating GEB colonization ability, genetic stability, and the mechanisms underlying their activities are still in their infancy, thereby requiring more basic and clinical studies to advance the clinical transition of GEBs.

#### Dose tolerance

Intensive studies have emphasized the key role of dose in the therapeutic effects of GEBs against diseases (Ott et al., 2004). *Bifidobacterium infantis* 35,624, which is generally used to treat irritable bowel syndrome, exhibited the best performance at a dose of 10^8^ cfu per day, while 10^6^ cfu and 10^10^ cfu per day did not result in significant differences from the placebo group (Whorwell et al., 2006), thus indicating that the use of bacteria against disease is closely related to the bacterial number. Although a high amount of bacteria generally exhibits enhanced therapeutic effects, potential side effects also increase (Leventhal et al., 2020). The oral administration of SYNB1020 at a concentration of 10^12^ cfu per person induced adverse reactions, such as nausea and vomiting, in healthy volunteers and hyperammonemia patients (Kurtz et al., 2019). Some studies further demonstrated that an increasing number of bacteria generally leads to the development of dose-dependent problems and may even restrain immune system memories (Sivick et al., 2018). Therefore, how to achieve better therapeutic effects with low doses of GEBs remains a hot topic in the development of therapeutically engineered bacteria. Increasing their targeting ability is a selective strategy to decrease the administered dose. For example, the expression of tumor-targeting adhesins on the membrane surface of *E. coli* significantly decreased the intravenous injected bacterial number required for the minimum effective dose (Piñero-Lambea et al., 2015).

Simultaneously, different administration methods also influence the effective doses of GEBs in disease treatment. *Lactobacilli* were administered *via* intraperitoneal injection, intestinal administration, and oral administration, but the doses required to reach the same therapeutic efficacy differed by nearly 10,000 times among them (Steidler et al., 2000). Notably, each bacterium has an optimal dose for producing the best therapeutic efficacy. The recommended dose for *Bifidobacterium infantis* 35,264 is 10^8^ CFU per day, while the optimal dose for probiotic preparation VSL#3 (VSL Pharmaceuticals) is 1.8 × 10^12^ CFU per day when taken by oral administration (Gionchetti et al., 2003). Although daily probiotic use has a long history, the use of bacteria for the prevention and treatment of disease is still at its initial stage.

#### Safety evaluation

Using bacteria to improve intestinal function and treat diseases has been proven to be a safe and effective modality (Sharif et al., 2017). However, GEBs prepared by genetic modification, chemical capsulation or other methods need to be fully evaluated for metabolic pathways and toxicological effects *in vivo* before clinical application. The pathogenicity of GEBs is mostly derived from the bacteria, but this defect could be partly removed or mitigated by the gene knockout or mutation of virulence genes. Typical examples include lipopolysaccharide deletion in gram-negative bacteria, virulence elimination in *Listeria monocytogenes*, and exotoxin gene knockout in *Clostridium novyi* (Zhou et al., 2018).

Additionally, the regulation of bacterial population provides another strategy to further enhance the safety of bacteriotherapy. A synchronous lysis circuit consisting of positive and negative feedback genes, and an inducible promoter can precisely control the proliferation and lysis of bacteria, thus restraining the bacterial population within a specified scope (Din et al., 2016). Moreover, population competition between the intrinsic bacterial flora and orally administered GEBs is always inevitable. Once foreign bacteria dominate the original flora, they probably alter the physiology of the host, thus leading to dysfunction of the gastrointestinal tract, such as inflammation (Spees et al., 2013) and pathogenic infection (Kamada et al., 2013). Therefore, purging foreign bacteria from the host after treatment is another key issue in GEB application. Biocontainment strategies, alternative selection markers and the use of homologous DNA have been performed to inhibit potential transmission in the environment and purge residual foreign bacteria (Plavec and Berlec, 2020). For instance, SYNB1618 must rely on exogenous diaminopimelate for cell wall synthesis in the case of dapA gene deletion, thus ensuring the complete purge of the bacteria after treatment (Isabella et al., 2018).

## GEB preclinical and clinical applications in disease management

As early as 100 years ago, bacteria were used to treat tumors and clostridial enteritis (Coley, 1893; Hoffman, 2016). Engineered bacteria at their initial stages mainly focused on the treatment of gastrointestinal inflammation and tumors (Steidler et al., 2000; Geirnaert et al., 2017; Zhou et al., 2018). Advances in genetic technology endowed GEBs with more functions and broad application prospects. An alternative method is to deliver foreign therapeutic drugs, thus alleviating the shortcomings of natural drugs in the low production level, short action time, and nonoral administration property. In this paragraph, we conclude and describe the use of GEBs for treating multiple diseases, such as IBD, obesity, diabetes and cancer, in detail (Table 1).

**Table 1:** Use of GEBs in disease treatment.

**Chassis cells**	**Therapeutic payload**	**Diseases**	**Animal experiment**	**Development stages**	**References**
*Lactobacillus*	IL-10	IBD	Yes	Mice	Steidler et al., 2000
*Lactobacillus*	IL-4	IBD	Yes	Mice	Souza et al., 2016
*E. coli*	IL-35	IBD	Yes	Mice	Zhang et al., 2018
EcN	Trefoil factor	IBD	Yes	Mice	Pedrolli et al., 2019
*Bacillus thermophilus*	Superoxide dismutase	IBD	Yes	Mice	Del Carmen et al., 2014
*Bifidobacterium*	RhMnSOD	IBD	Yes	Mice	Liu et al., 2018
*Lactobacillus*	Elafin	IBD	Yes	Mice	Bermúdez-Humarán et al., 2015
*Lactobacillus*	Recombinant mouse heme oxygenase-1	IBD	Yes	Mice	Shigemori et al., 2015
NZ9001					
*Lactobacillus*	Pancreatitis-related protein	Intestinal mucositis	Yes	Mice	Carvalho et al., 2017
EcN	Butyrate	Colon cancer HT29	Yes	Mice	Chiang and Hong, 2021
*Salmonella, Typhimurium*	IL-1β	Colon cancer	Yes	Mice	Kim et al., 2015
		CT26			
*E. coli*	Β-glucuronidase	Colon cancer	Yes	Mice	Afkhami-Poostchi et al., 2020
*Salmonella Typhimurium,*	Autoinducer	Colorectal cancer	Yes	Mice	Din et al., 2016
		MC26			
*EcN*	Tum-5	Melanoma	Yes	Mice	He et al., 2017
*Salmonella* VNP20009	Sox2	Lung cancer	Yes	Mice	Zhao et al., 2016
*Salmonella*	Transforming growth factor alpha-*pseudomonas* exotoxinTGFa-PE38	Colon cancer CT26 & Breast cancer 4 T-1	Yes	Mice	Lim et al., 2017
*Salmonella* SL7207	Diaminopimelate DAP	Hepatocellular carcinoma	Yes	Mice	Li et al., 2017
*Lactobacillus*	GLP-1	Diabetes	Yes	Mice	Duan et al., 2015; Lin et al., 2016
*Lactobacillus*	Heat shock protein 65HSP65, IA2P2	Diabetes	Yes	Mice	Liu et al., 2016
*Lactobacillus*	GLP-1	Obesity	Yes	Mice	Wang et al., 2021
*Bacillus subtilis* SCK6	Butyric acid	Obesity	Yes	Mice	Bai et al., 2020
*Bacillus subtilis* SCK6	BA	Obesity	Yes	Mice	Bai et al., 2020
EcN SYNB1020	I-arginineI-arg	HyperammonemiaHA	Yes	Stop	Kurtz et al., 2019
EcN SYNB1618	Insert phenylalanine ammonia lyase and L-amino acid deaminase gene	PhenylketonuriaPKU	Yes	Phase 1/2a	Isabella et al., 2018; Puurunen et al., 2021
*Lactobacillus plantarum*	Angiotensin-converting enzyme inhibitory peptidesACEIPS	Hypertensive	Yes	Mice	Yang et al., 2015
*Vibrio cholerae strain Haiti V*	Delete CTXF, CTXA, RECA genes	Cholera	Yes	Infant rabbit	Hubbard et al., 2018
*Meningitis MenB* YH102， YH103	Delete rfaF, metH, siaD	Meningitis	Yes	Mice	Li et al., 2004
EcN	Insert Phl p1 and Phl p5 gene, control the level of IgE	Allergic poly-sensitization	Yes	Mice	Sarate et al., 2019

### Gastrointestinal disease

The dysregulation of intestinal microbes induces gastrointestinal diseases and, in some cases, damages normal organs *via* the tissue-gut axis (Fassarella et al., 2021; Lee and Chang, 2021). Routine supplementation with probiotics successfully improved or cured types of diseases, such as acute diarrhea (Mu and Cong, 2019), IBD (Saez-Lara et al., 2015; Jakubczyk et al., 2020), and diabetes (Kobyliak et al., 2016; Razmpoosh et al., 2016; Kocsis et al., 2020; van de Wijgert and Verwijs, 2020; Davidson et al., 2021). Specifically, IBD, as an autoimmune disease, is characterized by chronic inflammation of the gastrointestinal tract and the loss of epithelial barrier integrity in the intestine (Fakhoury et al., 2014). Ulcerative colitis and Crohn’s disease are both classified as IBD. IBD has a high incidence worldwide, but there is still no efficient treatment method. Considering the key role of microorganisms in the intestinal microecological balance and IBD inflammatory characteristics, an anti-inflammatory cytokine, interleukin-10, was expressed in *Lactobacillus* to treat chronic disease, which finally led to a 50% therapeutic efficacy in a mouse IBD model (Steidler et al., 2000). Similarly, interleukin-4 (IL-4)-expressing *Lactobacillus* also significantly alleviated inflammatory responses caused by increased Th1 cells (Souza et al., 2016). Additionally, the oral administration of interleukin-35 (IL-35) expressing *E. coli* obviously attenuated inflammatory damage in mouse colon tissue, thereby improving the symptoms of IBD (Zhang et al., 2018). Furthermore, EcN has a long history in treating intestinal tract diseases in infants and toddlers. Using EcN to colonize and secrete intestinal trefoil factor could significantly improve the integrity of the intestinal epithelium and reduce the dextran sodium sulfate-induced intestinal inflammatory response in a mouse model (Pedrolli et al., 2019).

Moreover, another potential way to treat colitis is to increase superoxide dismutase or catalase in the intestine, thereby reducing reactive oxygen species, which is a key factor in inflammation (Hwang et al., 2020; Wan et al., 2022). Utilizing GEBs to produce foreign proteins to enhance the delivery of superoxide dismutase or catalase to the intestine successfully reduced the inflammatory reaction in a trinitrobenzene sulfonic acid-induced colitis model (Del Carmen et al., 2014). Additionally, the expression of manganese superoxide dismutase in *Bifidobacterium* greatly decreased dextran sodium sulfate-induced IBD (Liu et al., 2018). Furthermore, elastin is generally used to treat IBD because of its inhibitory effects on serine protease activity and anti-inflammatory effects (Bermúdez-Humarán et al., 2015). The oral administration of elastin-expressing *Lactobacillus* strains exhibited a strong inhibitory effect on dextran sodium sulfate-induced IBD (Bermúdez-Humarán et al., 2015). Similarly, genetically modified *Lactobacillus* strains could use mucosa to deliver an anti-inflammatory molecule, recombinant mouse heme oxygenase-1, and thus reduce the incidence of acute colitis (Shigemori et al., 2015). In addition to IBD, GEBs have been used to express foreign proteins, such as antibacterial pancreatitis-related protein, to alleviate or treat chemotherapy-induced intestinal diseases and mucositis in mice (Carvalho et al., 2017).

### Cancer

The specific tumor microenvironment enables the colonization of facultative anaerobic or anaerobic bacteria in the hypoxic regions of solid tumors. The use of tumor-targeting bacteria, such as *Bifidobacterium* (Chen et al., 2021), *Salmonella* (Chirullo et al., 2015; Kim et al., 2015; Li et al., 2017), *Clostridium novyi-NT* (Roberts et al., 2014; Staedtke et al., 2016; Janku et al., 2021), and *E. coli* (Afkhami-Poostchi et al., 2020; Chiang and Hong, 2021), to deliver various proteins, chemical molecules, preenzymes, etc. for cancer therapy is collectively referred to as bacteriolytic therapy. Detailing all anticancer GEBs is beyond the scope of this review, and this topic has been recently reviewed by others (Badie et al., 2021; Huang et al., 2021; Kalia et al., 2021).

### Metabolic diseases

The increasing prevalence of obesity, diabetes and other metabolic diseases in modern society places a heavy burden on medical care. These metabolic diseases are generally characterized by hyperglycemia, hyperlipidemia and high-density lipoprotein, and simultaneously, they could trigger each other (Guo, 2014). Notably, most patients with metabolic diseases must take drugs for the rest of their lives to alleviate or slow disease progression. In contrast, the oral administration of GEBs has exhibited few side effects and better acceptance. Indeed, many GEBs have already been constructed and evaluated for curing inborn or acquired metabolic diseases.

#### Diabetes

Diabetes is one of the greatest public health problems worldwide and causes a substantial burden on the socioeconomic development of the world (Lin et al., 2020). Diabetes is clinically divided into type 1 diabetes (T1D) and type 2 diabetes (T2D; Zimmet et al., 2014). The treatment for T1D relies on the regular injection of insulin to slow the progression of the diabetic process (Miller et al., 2015), whereas T2D patients take hypoglycemic drugs, such as metformin, to restrain disease progression. However, the long-term use of glucose-lowering drugs usually results in serious side effects and economic burdens (Khunti et al., 2019). Numerous studies have found that disease progression in T2D is closely associated with an imbalance in gut microflora (Forslund et al., 2015). Therefore, the regulation of the intestine microenvironment using probiotics or GEBs could be an alternative strategy to cure or slow diabetes, especially T2D. Given the underlying mechanisms of diabetes, treatment could be achieved by using an engineered bacterium to produce proteins with hypoglycemic effects. For example, glucagon-like peptide 1 (GLP-1) is an efficient drug for diabetes treatment, but its short half-life time and high cost greatly prevent its extensive clinical use. However, GEBs characterized by gut colonization have the potential to address the above problems. Leveraging GLP-1 (1–37) with the ability to transfer intestinal epithelial cells into insulin-secreting cells (Duan et al., 2015), GLP-1-expressing *Lactobacillus* successfully normalized blood glucose levels in diabetic Goto-Kakizaki rats (Lin et al., 2016). Other proteins or compounds against diabetes can also achieve similar therapeutic effects by using probiotics as delivery vectors. For example, recombinant *Lactobacillus* expressing heat shock protein 65 and IA2P2 (a 23 amino acid peptide) effectively alleviated the symptoms of pancreatitis and improved diabetes by inhibiting the antigen-specific proliferation of T cells in T1D and regulating the balance between Th17/Tregs and Th1/Th2 cells (Liu et al., 2016).

#### Obesity

Obesity is a complex multifactorial disease and a key factor in other chronic diseases, such as cardiovascular disease, cancer, and diabetes (Pan et al., 2021). Generally, obesity occurs in middle-aged and elderly people. Increasing reports demonstrate that the prevalence of obesity in children and adolescents aged 2 to 19 years has been gradually increasing worldwide, especially in developed countries (Fryar et al., 2018, 2020). Most traditional anti-obesity drugs, such as sibutramine and orlistat, act by suppressing appetite or blocking the absorption of body fat, but they produce obvious adverse effects on normal physiological function (Huang et al., 2019). Fortunately, several studies have developed GEBs to express anti-obesity factors for the purpose of alleviating obesity. For instance, GLP-1-expressing *Lactobacillus* could reduce the incidence of high-fat diet-induced obesity in mice by restraining lipid accumulation, enhancing GLP-1 resistance to glucose intolerance, and increasing the expression of genes involved in the triglyceride degradation pathway (Wang et al., 2021). Additionally, *Bacillus subtilis* SCK6 utilized Coenzyme A transferase acetate to increase butyric acid production and stimulate the butyric acid kinase pathway, thus attenuating hepatic steatosis and fat accumulation in high-fat diet mice (Bai et al., 2020). Similarly, interleukin-22 (IL-22)-expressing *Lactobacillus reuteri* obviously decreased the incidence of nonalcoholic fatty liver disease in high-fat diet mice (Oh et al., 2020).

#### Hyperammonemia, phenylketonuria and other metabolic diseases

Hyperammonemia, as a metabolic disease, is used to describe patients with high levels of plasma ammonia levels (>50 μmol/l in adults and >100 μmol/l in the neonatal period). Under normal circumstances, ammonia is mainly produced in the intestine and excreted by the liver, but severe metabolic disorders probably induce the massive accumulation of ammonia in the body and, thus, affect the urea cycle and may even trigger hepatic encephalopathy (Auron and Brophy, 2012). The current treatment strategies for patients with hyperammonemia include hemodialysis, peritoneal dialysis, and antibiotic therapy. However, these treatments greatly increase infection probabilities and lead to drug resistance (Matoori and Leroux, 2015). However, oral probiotic use could significantly decrease ammonia levels in mice with hepatic encephalopathy (Lunia et al., 2014), demonstrating the feasibility of establishing GEBs to treat Hyperammonemia. In addition, by deleting the negative regulator of I-arginine synthesis and adding a feedback-resistant I-arginine biosynthetic enzyme in EcN, GEBs successfully achieved the conversion of NH3 to I-arginine in bacteria, thereby blocking ammonia accumulation *in vivo* and slowing Hyperammonemia progression (Kurtz et al., 2019).

Phenylketonuria is an autosomal recessive genetic disease characterized by mental and growth retardation. Phenylketonuria results from the gene deficiency of phenylalanine hydroxyls, thus preventing the conversion of phenylalanine to tyrosine. The Phenylketonuria therapeutic methods include dietary restriction, gene therapy, and enzyme replacement. However, even a well-controlled diet cannot completely prevent the occurrence of psychiatric problems in Phenylketonuria patients, while gene therapy is extremely costly, and enzyme replacement therapy is obviously affected by the drug dose and administration schedule (Camp et al., 2014). However, probiotics have the potential to be modified to produce deficient phenylalanine hydroxyl enzymes to supplement physiological requirements. The insertion of the genes of phenylalanine-ammonia-lyase and L-amino acid deaminase into the EcN genome enables probiotics to convert phenylalanine into trans-cinnamic acid salt in the gastrointestinal tract, thus leading to a 38% reduction in orally administered phenylalanine in blood (Isabella et al., 2018; Puurunen et al., 2021).

Furthermore, hypertension is a common chronic disease that is not only a major cause of cardiovascular disease but also damages the brain, kidney, and other organs (Fuchs and Whelton, 2020). Many therapeutic agents, including β-blockers, calcium channel blockers, diuretics, and renin-angiotensin converting enzyme inhibitors, have been developed to treat hypertension. However, the long-term use of these antihypertensive drugs often leads to potential side effects, such as arterial damage, angioedema, arrhythmias, impotence, hyperkalemia, and cough. More importantly, some drugs, such as angiotensin-converting enzyme inhibitory peptides, require large doses to exert therapeutic effects because their activity is inclined to be inhibited by other enzymes (Laurent, 2017). However, probiotics engineered to express the enzyme have the potential to address this issue. For example, the introduction of the genes encoding tuna frame protein and yellowfin sole frame protein into *Lactobacillus plantarum NC8* enabled the probiotics to synthesize angiotensin-converting enzyme inhibitory peptides, and the oral administration of angiotensin-converting enzyme inhibitory peptides significantly reduced systolic blood pressure and triglyceride levels in a spontaneously hypertensive mouse model, displaying good antihypertensive ability (Yang et al., 2015).

## Conclusion and prospects

Microbes have been implicated in almost all fundamental activities of *physiological homeo*stasis. One example of the increasingly important role of bacteriotherapy is the regulation of the intestinal flora for curing intestinal diseases and adjuvant therapy of other diseases, such as cancer and metabolic diseases (Andrade-Oliveira et al., 2015; Alexander et al., 2017; Yuan et al., 2018; Molina et al., 2021; Si et al., 2021). Notably, advances in gene sequencing technologies have partly revealed the mysteries of human microbes and the interaction between microbes and various mammalian cells, greatly advancing GEB preclinical studies. To date, most existing therapeutic GEBs have been modified from human intestinal probiotics, demonstrating their potential safety compared to traditional chemical drugs, especially for long-term use. GEBs are not only effective therapeutic agents against chronic diseases due to their intestinal colonization ability but also expand the scope of the application of tumor-targeting treatment.

The use of engineered bacteria in disease treatment is still in the infancy stage and has limitations for incurable diseases. In addition, the clinical translation of GEBs is still hindered by potential pathogenicity and local laws and regulations, which only allow clinical trials to use bacteria without any genetic modification. A future challenge will be to determine whether the genetically inserted genes of GEBs could spill over into the genomes of other bacteria or mammalian cells; how GEBs could stably colonize and produce the expected substrates in the targeted sites; how they interact with the intestinal flora, thus normalizing them into the health status; and how to lock them into the expected tissues and clear them once they accomplish their mission. Such questions underscore the importance of investigating the genetic stability of the inserted genes in GEBs under normal physiological conditions. However, as biological technologies continue to evolve, newly available bacterial tools and the upgrading of therapeutic strategies of GEBs will further alleviate potential safety concerns and enhance their depth and breadth in disease prevention and treatment.

Collectively, given the importance of the microbiota in normal physiological function, the long historical use of probiotics and bacterial-derived products for health care, and our increasingly better understanding of the mechanisms underlying the various gut-brain similar axes, we expect that GEBs will make an important contribution to the prevention and treatment of various diseases that current drugs cannot address or cause serious side effects.

## Author contributions

YL, JF, and HP searched references and drafted the manuscript. XZ and YZ conceived, supervised, and improved the manuscript. All authors contributed to the article and approved the submitted version.

## Funding

This research was supported by the National Natural Science Foundation of China (grant no. 81971726) and the Nanjing Healthcare Science and Technology Development Special Funded Project (YKK20191).

## Conflict of interest

The authors declare that the research was conducted in the absence of any commercial or financial relationships that could be construed as a potential conflict of interest.

## Publisher’s note

All claims expressed in this article are solely those of the authors and do not necessarily represent those of their affiliated organizations, or those of the publisher, the editors and the reviewers. Any product that may be evaluated in this article, or claim that may be made by its manufacturer, is not guaranteed or endorsed by the publisher.
